# Antiphospholipid Antibody Assays in 2021: Looking for a Predictive Value in Addition to a Diagnostic One

**DOI:** 10.3389/fimmu.2021.726820

**Published:** 2021-09-21

**Authors:** Pier Luigi Meroni, Maria Orietta Borghi

**Affiliations:** ^1^Istituto Auxologico Italiano, IRCCS, Immunorheumatology Research Laboratory, Milan, Italy; ^2^Department of Clinical Science and Community Health, University of Milan, Milan, Italy

**Keywords:** thrombosis, miscarriages, antiphospholipid antibodies, β_2_-glycoprotein I, prothrombin

## Abstract

Antiphospholipid antibodies (aPL) are mandatory for the diagnosis but are also a risk factor for the antiphospholipid syndrome (APS) clinical manifestations. Lupus anticoagulant (LA), anticardiolipin (aCL), and anti-beta2 glycoprotein I (β_2_GPI) assays are the formal laboratory classification/diagnostic criteria. Additional nonclassification assays have been suggested; among them, antiphosphatidylserine-prothrombin (aPS/PT) and antidomain 1 β_2_GPI antibodies are the most promising ones although not yet formally accepted. aPL represent the example of a laboratory test that moved from dichotomous to quantitative results consistent with the idea that reporting quantitative data offers more diagnostic/prognostic information for both vascular and obstetric manifestations. Although the general rule is that the higher the aPL titer, the higher the test likelihood ratio, there is growing evidence that this is not the case for persistent low titers and obstetric events. LA displays the highest diagnostic/prognostic power, although some isolated LAs are apparently not associated with APS manifestations. Moreover, isotype characterization is also critical since IgG aPL are more diagnostic/prognostic than IgA or IgM. aPL are directed against two main autoantigens: β_2_GPI and PT. However, anti-β_2_GPI antibodies are more associated with the APS clinical spectrum. In addition, there is evidence that anti-β_2_GPI domain 1 antibodies display a stronger diagnostic/prognostic value. This finding supports the view that antigen and even epitope characterization represents a further step for improving the assay value. The strategy to improve aPL laboratory characterization is a lesson that can be translated to other autoantibody assays in order to improve our diagnostic and prognostic power.

## Introduction

The antiphospholipid syndrome (APS) is formally defined as the association of arterial/venous thrombosis and/or recurrent miscarriages in the absence of any other known cause and the persistent presence of antiphospholipid antibodies (aPL) detectable by solid-phase (beta2 glycoprotein I [β2GPI]-dependent anticardiolipin [CL] and anti-β2GPI) or functional coagulation assays (lupus anticoagulant—LA) (**Table 1**) (1). Additional laboratory diagnostic tests have been suggested, but their formal inclusion in the classification tools is still a matter of debate (**Table 1**) (1, 2). The detection of aPL represents a milestone in the diagnosis of APS despite the still debated description of rare seronegative APS in which the clinical manifestations are resembling the full-blown syndrome, but the serological assays are negative (3).

**Table 1:** Classification and nonclassification laboratory aPL assays.

Target Ag	Plates coated with/biological material used	Technical characteristics of the assay and type of detectable antibodies
Bovine β_2_GPI	Anionic PL	aCL solid phase assay
Human β_2_GPI	γ-irradiated plates	Anti-β_2_GPI solid phase assay
Human β_2_GPI/Domain I/Domain I peptide	Hydrophobic/hydrophilic or γ-irradiated plates	Anti-DI β2GPI solid phase assay
Human PT	Anionic PL (PS)	Anti-PT/anti-PS/PT solid phase assay
Protein C, Protein S and C4b-binding protein Activated Protein C Thrombomodulin	Anionic PL	Mostly anti-β2GPI antibodies
Annexin V	Anionic PL	Mostly anti-β2GPI antibodies
High molecular weight kininogen	Neutral PL (PE)	Anti-PE solid phase assay
Human β_2_GPI/PT	Human plasma	LA: functional PL-dependent coagulation assay

β_2_GPI, beta2 glycoprotein I; PL, phospholipids; aCL, anticardiolipin antibodies; PT, prothrombin; PS, phosphatidylserine; PE, phosphatidylethanolamine; LA, lupus anticoagulant.

There is strong evidence that aPL, rather than being a mere diagnostic tool, display a direct pathogenic role through complement-fixing antibodies in animal models (4). Medium/high titers of aPL detectable by solid-phase assays (i.e., aCL and anti-β2GPI) or the positivity for two or three laboratory assays confer a higher risk for both vascular and obstetric events than low titer aPL or positivity in a single test only (5, 6). Preliminary studies raised the issue of whether abnormalities in serum complement levels can be predictive for a poor pregnancy outcome, but confirmatory studies are still needed and to be extended to vascular APS (7, 8). So, aPL are emerging as a risk factor, and their high likelihood ratio/predictive value is becoming more and more important. This is actually in line with the similar need reported for other autoantibodies in systemic autoimmune rheumatic diseases (SARD) (9, 10).

How to interpret the aPL assays correctly and which assays should be requested for the best diagnostic/prognostic strategy are the main questions that will be addressed in the present mini-review to offer a state-of-the-art of aPL testing in 2021.

## Laboratory Perspectives

### Autoantibodies in Diagnostic and Classification Criteria for APS

The three aPL assays (i.e., β2GPI-dependent aCL, anti-β2GPI, and LA) are the formal classification laboratory tests that are also commonly used for diagnostic purposes (1).

In 1990, three different groups reported that aPL do not recognize anionic PL alone but bound to a PL-binding glycoprotein, later identified as β2GPI (11–13). The anti-β2GPI antibodies bind their antigen either when complexed with CL in the presence of a source of β2GPI in CL-coated plates or directly in β2GPI-coated plates. It has been suggested that once bound to CL, β2GPI displays conformational changes and/or increases its antigenic density so favoring antibody binding (5, 14). On the other hand, β2GPI coating to γ-irradiated polystyrene plates is thought to reproduce similar molecule presentation ultimately offering the right antigen structure to the antibodies (5, 14). In other words, β2GPI-dependent antibodies are responsible for positive results in the two solid-phase assays that are the formal laboratory classification criteria for APS, namely aCL and anti-β2GPI antibody tests.

The term “lupus anticoagulant” (LA) refers to a panel of different functional assays detecting a heterogeneous group of immunoglobulins behaving as acquired *in vitro* inhibitors of the coagulation. LA detection is based on PL-dependent coagulation tests requiring complex methods. The interpretation of the results is difficult owing to interfering factors, such as anticoagulant drugs and acute phase proteins leading to false-positive results (15–17). The International Society of Thrombosis and Haemostasis has recently provided the updated guidelines for LA detection/interpretation (18). Anti-β_2_GPI antibodies have been shown to prolong the PL-dependent coagulation tests and were thought to be responsible in part for the so-called LA phenomenon (19–21). This finding supports the idea that β_2_GPI-dependent aPL can be responsible for the positivities in all the three formal laboratory classification (and diagnostic) tests for APS. On the other hand, antibodies against prothrombin (aPT) and in particular those reacting with the phosphatidylserine (PS)-PT complex (aPS/PT) have been also shown to mediate the LA phenomenon (22–24). Finally, “isolated” LA without any anti-β_2_GPI or aPS/PT antibodies has been described. In these samples, the coagulation inhibitors (antibodies)? are still a matter of research (25, 26).

### Nonclassification Laboratory Criteria

Although both IgG and IgM aPL have been included in the laboratory classification criteria (1), the IgG isotype has displayed a higher diagnostic and prognostic value than the IgM one for both the vascular and the obstetric manifestations of the syndrome since the beginning of the APS story (27, 28). More recently, several groups suggested that IgA aPL may offer a good diagnostic/prognostic profile as well. This was the case in patients with clinical manifestations suggestive for APS but negative for aCL/anti-β2GPI IgG or IgM or LA (29–33). In particular, IgA aCL/anti-β_2_GPI positivities were reported in systemic lupus erythematosus (SLE) patients with associated APS (29–32). Therefore, the detection of IgA aPL is becoming more and more popular in the diagnostic algorithm for APS. However, IgA aPL are not formally included in the laboratory classification criteria yet (32).

The conformational modifications of the β2GPI are in line with the theory that most of the β2GPI-dependent aPL recognize an immunodominant epitope located in the domain (D)1 of the molecule. It has been suggested that β2GPI, once bound to anionic surfaces, undergoes structural changes making the D1 more available for the antibodies (14, 34). There is sound evidence that anti-D1 antibodies mediate pathogenic mechanisms in experimental models and support clotting and fetal loss in animal models (35–37). Moreover, clinical studies clearly showed that the presence of anti-D1 β2GPI IgG displays a higher specificity and predictive value than IgG against the whole molecule (38–41). Accordingly, anti-D1 detection has been suggested as a new laboratory criterion for APS (32, 42). However, up to 20% of the patients positive for antibodies against the whole β2GPI molecule can test negative for specific anti-D1 assays (32). As a consequence, the idea to replace the whole molecule solid-phase assay with the test for anti-D1 has not been accepted yet. However, the presence of antibodies against D1 has been suggested to be a sort of a confirmatory test for aPL specifically associated with APS. For example, anti-D1 antibodies are not usually detected in aPL present during infectious diseases (43, 44) or in other conditions unrelated to APS, such as in children with atopic dermatitis or babies born from mothers with non-APS autoimmune disorders (38).

Antibodies against linear epitopes of other β2GPI domains have been reported, but clear associations with specific clinical manifestations of the syndrome were not found (45). However, antibodies against a D4-5 conformational complex have been recently investigated in a deeper manner. These antibodies have been mostly detected in non-APS patients such as patients with aPL and concomitant infectious disease or in children suffering from atopic dermatitis or in babies born from mothers with SARD (38, 40, 41, 43, 44). Polyclonal IgG from subjects/patients positive for isolated anti-β2GPI D4,5 antibodies were not able to trigger thrombosis in naiїve rats at variance with anti-D1 polyclonal IgG that were thrombogenic in the same model (35).

Moreover, higher titers and prevalence of anti-D4,5 IgG were found in asymptomatic aPL-positive carriers (40, 41). Altogether these data strongly support the idea that anti-D4,5 antibodies are not pathogenic and not diagnostic for APS (46). Interestingly, anti-D4,5 antibodies mainly recognize D5 and react with β2GPI free in solution or with the molecule bound to γ-irradiated polystyrene plates but not with β2GPI bound to CL. Since D5 is located in the PL-binding site of β2GPI, it has been suggested that D5 is available when the molecule is free in solution or when the coating to the plates does not involve the PL-binding site. The engagement of D5 in the PL-binding site (e.g., through CL) would be responsible for a steric hindrance and ultimately for the lack of reactivity of the anti-D5 antibodies (35).

As stated before, the LA phenomenon can be also mediated by aPT antibodies. Solid- phase assays with a matrix coated with PT were set up and aPT antibodies were detected. However, these antibodies did not display a good diagnostic or predictive value for the APS clinical manifestations (47, 48). On the other hand, when PT binds to PS-coated plates in the presence of Ca ions, it displays a right conformational change and can be recognized by aPS/PT antibodies. These antibodies have been found associated with APS, and their presence may increase the diagnostic/prognostic value of the other antibodies (e.g., aCL/anti-β2GPI and LA) (48). This is the case of the so-called tetrapositive patients (49). While aPS/PT have been reported in vascular APS, their association with the obstetric manifestations is still a matter of research (50–52). So, the inclusion of aPS/PT antibodies into the formal laboratory classification criteria has not been formally accepted up to now (32). Since aPS/PT antibodies were found to be associated with LA, some authors suggested their use as a surrogate test for LA when the interference of the concomitant anticoagulant therapy cannot allow performing the functional assays in a reliable manner (53). While the debate to include aPS/PT antibodies into the laboratory classification criteria is open, the experimental evidence for a direct pathogenic role for aPS/PT is not as sound as that reported for the anti-β2GPI antibodies (2, 5).

Other anionic PL, such as PS or phosphatidic acid (PA) or phosphatidylinositol (PI), have been used to coat the matrix in order to substitute CL in alternative solid-phase assays. Once again, β2GPI, as a cationic molecule, forms a complex with the anionic PL and eventually offers similar antigenic targets for the antibodies. Accordingly, even PS- or PI- or PA-coated plates are actually detecting β2GPI-dependent antibodies, and there is no sound evidence that they offer further diagnostic information (5, 54).

Additional tests have been reported in the literature to detect antibodies directed against serum proteins that bind to anionic surfaces, such as Annexin V, Protein C (activated Protein C), and Protein S. All these tests are actually detecting antibodies against β2GPI, so it is not clear whether or not they are offering more diagnostic/prognostic information in comparison with the β2GPI assay itself (5, 55–58). Antibodies directed against high molecular weight kininogen bound to neutral PL such as phosphatidylethanolamine (PE) have been reported, but their usefulness is limited to a handful of cases with clinical manifestations similar to those present in APS (59).

### Standardization of aPL Assays

The comparability in performing and the uniformity in interpreting test results in the diagnostic algorithms for autoimmune diseases are hot issues because of the lack of harmonization despite their increasing use and the development of new techniques (9, 60). The same problem has been raised in APS given the huge variability of aPL results reported at the beginning of the APS story. The switch from enzymatic or fluorimetric solid-phase assays to chemiluminescent techniques improved the sensitivity without affecting the specificity and at the same time offering more reproducibility. Ultimately, the aPL detection methods available nowadays offer more reproducible results and allow harmonization as recently shown in a large multicenter study (61). Still, we have some unmet needs in the field of aPL testing. For example, the high sensitivity of the new assays raised the issue of a wide range of borderline results formally higher than the cutoff of healthy subjects but with doubtful clinical significance. A critical interpretation of the real diagnostic/prognostic value of borderline results is strongly recommended in the clinical setting, and operators are invited to perform their own cutoff values. While there is a general agreement that only medium/high aPL titers in the solid-phase assays should be taken into account to support the diagnosis of vascular APS, recent evidence is supporting the usefulness of low titer aPL in the obstetric variant (62).

As in the case of many other laboratory diagnostic tests for autoimmune diseases, we do not have international standards to express the test results in international units. However, the Committee on Harmonization of Autoimmune Testing of the International Federation of Clinical Chemistry and Laboratory Medicine in collaboration with the Joint Research Institute of the European Commission has studied the possibility of developing a certified reference material (CRM) with an assigned property value (anti-β2GPI IgG antibodies concentration in a matrix material). The availability of such CRM should offer the possibility to express the results in absolute values further improving the harmonization of aPL testing (63).

## Clinical Perspectives

### Clinical Significance for Vascular APS

As stated before, aPL are now generally accepted as a risk factor for the clinical manifestations of the syndrome. In particular, the probability of thrombotic recurrences in the vascular APS is correlated with the aPL titer, being medium/high antibody levels associated with arterial/venous events much more than low titers. Moreover, the simultaneous positivity for two or three classification laboratory tests is an additional risk factor for recurrences. More recently, it has been suggested that the presence of aPS/PT antibodies in addition to the three laboratory classification criteria (i.e., LA, aCL, anti-β2GPI) represents a further risk factor in the so-called tetrapositive patients (6, 49).

Antiphospholipid antibodies of the IgG Isotype display a more predictive value for the vascular manifestations in comparison with IgM. There is growing evidence that IgA aPL can be more predictive for vascular events than IgM as well; however, more data should support this statement (30, 31, 64).

Among the three formal classification laboratory assays, LA is widely considered the most predictive one, even if isolated LA positive cases can be found not associated with any vascular events (26, 49, 65, 66). The high predictive value of LA was related to the presence of both anti-β2GPI and aPS/PT antibodies in most of the LA positive samples (24, 67–69). Moreover, as a functional coagulation assay, LA displays a lower sensitivity compared with the solid-phase assays in detecting the same amount of autoantibodies. So, the higher aPL titers needed for altering the coagulation assay could justify the stronger predictive power for the clinical manifestations in both the full-blown APS and in the aPL-positive asymptomatic carriers.

Isolated aCL positive results, in particular at medium/low titer, are more frequently reported than isolated anti-β2GPI in non-APS conditions such as during concomitant infectious diseases. Their clinical significance is doubtful and should be evaluated in a specific clinical setting.

**Figure 1:**
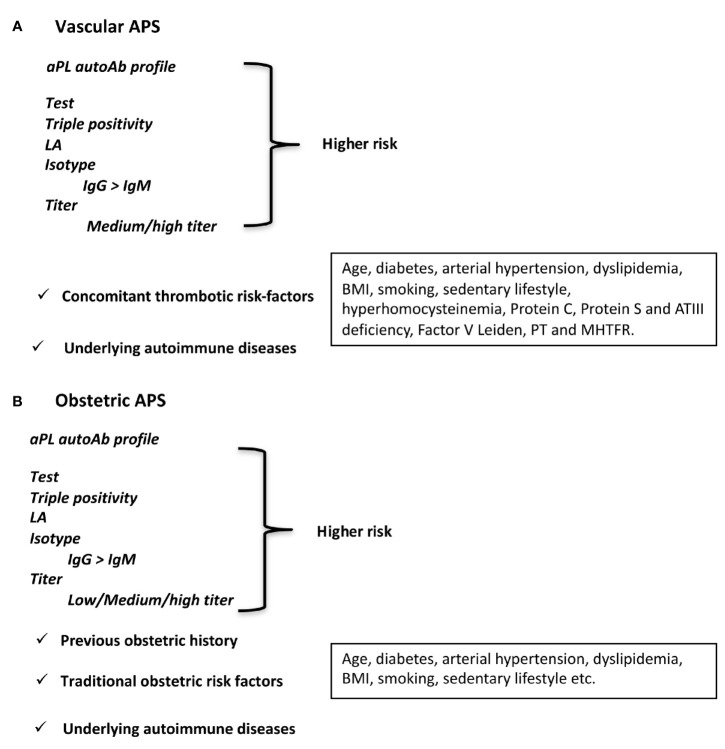
Antiphospholipid antibodies as a risk factor. aPL profile, isotype, titer, and aPL-unrelated factors defining higher risk for **(A)** vascular APS and **(B)** obstetric APS.

As shown in **Figure 1A**, the whole risk profile for the vascular APS is supported not only by the aPL profile (e.g., titer, isotype, type of the detection assay) but also by aPL-unrelated variables such as traditional cardiovascular risk factors and the presence of an associated underlying SARD. In particular, the association with a systemic inflammatory disease may offer a significant trigger according to the two-hit hypothesis for APS (5).

### Clinical Significance for Obstetric APS

Medium/high aPL titers and double or triple positivity for the classification laboratory criteria do represent the major risk factor for the obstetric manifestations of APS as for the vascular ones. However, it has been suggested that even low aPL titers can display a prognostic value for recurrent miscarriages (62, 70, 71). This issue has been addressed recently by a large monocentric study that showed how the positivity for aCL and anti-β2GPI, if persistent over time and associated, may be predictive for miscarriages. The finding is also important from a clinical point of view since all the low titer pregnant women were responsive to the standard therapy with the combination of LDASA and LMWH at variance with patients with medium/high aPL titers who display recurrences in up to 20% of the cases (62). The demonstration of the huge presence of β2GPI in the placenta, even in physiological conditions, could explain why low aPL titers may be enough for displaying their pathogenic effect. This is not the case for β2GPI on the vessel walls in resting conditions where the aPL target antigen cannot be found unless an endothelial perturbation is taking place. The lower presence of β2GPI on the vessels could explain, on the other hand, why much higher amounts of aPL are needed for triggering the clot (71, 72).

A similar higher risk profile of the IgG than IgM isotype for aCL and anti-β2GPI assays and the more predictive value of isolated LA in comparison with isolated aCL or anti-β2GPI test have been reported for the recurrent miscarriages as well (41, 70).

As for vascular APS, the whole risk profile for obstetric variant should take into consideration additional aPL-unrelated risk factors such as the previous obstetric history and/or the presence of an underlying systemic autoimmune inflammatory disorder (**Figure 1B**) (71).

### Asymptomatic aPL-Positive Carriers

As discussed before for patients with both the full-blown vascular and obstetric syndrome, the aPL profile is crucial to characterize the risk for APS manifestations even in subjects with positive aPL but without any previous thrombotic event or miscarriage: the so-called asymptomatic aPL-positive carriers. The risk of these subjects for developing clinical events is likely similar to that in APS patients, but there are a few *ad hoc* prospective studies to support it in a formal way (26, 73). In summary, the presence of a double or triple positivity for the classification laboratory criteria, the medium/high aPL titer in the solid-phase assays, the positivity for IgG/IgA *versus* IgM antibodies, and the epitope specificity for D1 of anti-β2GPI are the parameters useful for risk stratification.

The presence of aPL-unrelated traditional cardiovascular or obstetric risk factors can play an additional role in the risk profile as also previously discussed for APS patients (**Figure 1**). Unfortunately, we still do not have sound information on which type of therapeutic intervention is the best to prevent clinical manifestations. *Ad hoc* clinical trials should be carried out.

### Is There a Value of Repeated Autoantibody Testing in Symptomatic At-Risk Patients?

Antiphospholipid antibodies are persistent over time according to the classification criteria of the syndrome (1). There is no sound evidence that they can fluctuate for example during an acute thrombotic event or during pregnancy. In this regard, aPL are quite similar to other autoantibodies detectable in SARD, such as rheumatoid factor, anticitrullinate peptide antibodies, or antibodies against extractable nuclear antigens. Nevertheless, a decrease in the aPL titer has been reported in some cases during a long follow-up, especially in patients receiving treatment with antimalarials (hydroxychloroquine) and/or anti-B cell therapy (anti-Blys monoclonal antibody) (74–76). On the other hand, transient positivities are usually described for aPL detectable in non-APS conditions, in particular during infectious diseases (77). As a consequence, repeated aPL testing is suggested for confirming the positivity and to support the suspect that the antibodies are related to a concomitant infectious disease but not for monitoring the classical APS.

## Discussion

The right choice and interpretation of the diagnostic aPL assays are pivotal to avoid the risk of an overdiagnosis, having in mind that both thrombosis and miscarriages are relatively frequent and due to several causes unrelated to aPL. For example, low aPL titers, isolated positivities in one single laboratory test, as well as transient positivities should be critically evaluated. Anti-β2GPI antibodies with D4,5 specificity are positive in the anti-β2GPI but negative in the aCL assay as reported previously. These antibodies are not associated with APS manifestations and are not pathogenic in animal models; altogether this finding supports that they are not diagnostic aPL (35). Another example is represented by isolated LA positivities in patients under heparin or oral anticoagulation that can affect the reproducibility of the test. High levels of C reactive protein have been also associated with false LA results, especially in patients during acute illness (15–17). So, positive LA tests in these conditions should be critically evaluated before making a final diagnosis. The use of solid-phase assays for antibodies potentially responsible for LA, such as β2GPI and aPS/PT, could help since the solid-phase assays are not affected by the variables responsible for false-positive functional tests (61).

Nonclassification laboratory tests such as antidomain assays or the test for aPS/PT could help in ruling out or in supporting the diagnosis of APS. For example, the lack of reactivity against D1 in a single positive anti-β2GPI patient or the negativity for aPS/PT in an isolated LA during anticoagulation cast doubts on the real presence of an APS. The strategy of using a panel of biomarkers (e.g., different autoantibodies) is becoming more and more popular in APS as well as in other autoimmune diseases and meets the need of precision medicine in this setting.

## Author Contributions

PM drafted the text and MB contributed to the article. Both authors revised and approved the manuscript.

## Funding

The study was supported in part by Ricerca Finalizzata, Ministero Salute 2020 to PM.

## Conflict of Interest

The authors declare that the research was conducted in the absence of any commercial or financial relationships that could be construed as a potential conflict of interest.

## Publisher’s Note

All claims expressed in this article are solely those of the authors and do not necessarily represent those of their affiliated organizations, or those of the publisher, the editors and the reviewers. Any product that may be evaluated in this article, or claim that may be made by its manufacturer, is not guaranteed or endorsed by the publisher.
